# Recent Advances of Transition Metal Chalcogenides as Cathode Materials for Aqueous Zinc-Ion Batteries

**DOI:** 10.3390/nano12193298

**Published:** 2022-09-22

**Authors:** Ying Liu, Xiang Wu

**Affiliations:** School of Materials Science and Engineering, Shenyang University of Technology, Shenyang 110870, China

**Keywords:** aqueous zinc ion battery, transition metal chalcogenides, layered structure, cathode, energy storage mechanism

## Abstract

In recent years, advances in lithium-ion batteries (LIBs) have pushed the research of other metal-ion batteries to the forefront. Aqueous zinc ion batteries (AZIBs) have attracted much attention owing to their low cost, high capacity and non-toxic characteristics. Among various cathodes, transition metal chalcogenides (TMCs) with a layered structure are considered as suitable electrode materials. The large layer spacing facilitates the intercalation/de-intercalation of Zn^2+^ between the layers. In this mini-review, we summarize a variety of design strategies for the modification of TMCs. Then, we specifically emphasize the zinc storage capacity of the optimized electrodes. Finally, we propose the challenges and future prospects of cathode materials for high-energy AZIBs.

## 1. Introduction

The growing energy crisis has driven the unprecedented development of renewable clean energy [1,2,3]. To date, lithium-ion batteries (LIBs) are the most widely used energy storage devices. However, the scarcity of lithium resources, the inflammability of electrolytes, and high operating environment requirements limit their growth [4,5,6,7]. Rechargeable aqueous zinc ion batteries (AZIBs) are a new generation of safety batteries. They possess certain advantages in terms of abundant zinc reserves, low anode potential (−0.763 V vs. SHE) and high theoretical capacity (820 mAh g^−1^) [8,9,10,11]. Therefore, AZIBs have become one of the candidates to replace LIBs. However, zinc anodes undergo dissolution–precipitation reactions with several adverse reactions, such as dendrite growth, corrosion, and by-product formation. They are inevitable during repeated plating and stripping and seriously damage the cycle life of the cells [12]. Also, divalent zinc ions possess stronger electrostatic interactions than monovalent lithium ions [13,14]. Therefore, the choice of a suitable intercalation material is the crucial to break through this challenge.

In recent years, many efforts are devoted to the exploration of cathode materials, including Prussian blue analogs, vanadium-based and manganese-based compounds, and transition metal chalcogenides (TMCs) [15,16,17,18,19]. Among them, Prussian blue analogs are featured by a high voltage window, but their crystal structure is unstable and prone to phase transformation [20]. The inherent low electrical conductivity and poor structural stability of V- and Mn-based materials lead to their slow electrochemical kinetics [21,22,23]. The electrical conductivity of TMCs is superior to that of oxides. Additionally, TMCs are characterized by a unique layer structure with large layer spacing. Their high specific surface area can provide many active sites and reduce ion transfer paths [24]. In previous reports, Naveed et al. designed the VS_2_ nanosheet materials as cathodes, which maintain a capacity of 138.3 mAh g^−1^ at 0.1 A g^−1^ after 500 cycles with a retention of 94.38% [25]. Kang’s group summarized the Zn storage ability of various oxides, sulfides and borides [26]. The results show that the activated MnS electrode is a potential cathode material with both high capacity and stable cycling performance. However, bulk MoS_2_ and WS_2_ materials are virtually incapable of storing zinc ions. Therefore, it is essential to improve the electrochemical performance of TMCs by effective tuning strategies.

In recent years, there have been numerous reports on TMCs-based cathode materials. Herein, we first summarize several feasible strategies for optimizing the electrode structure. Then, we discuss the electrochemical performance of TMCs cathodes for AZIBs. Lastly, we overview the advances in cathode materials and present the current challenges and prospects for constructing advanced cathodes of AZIBs.

## 2. The Electrochemical Performance of TMCs Cathodes

**Figure 1:**
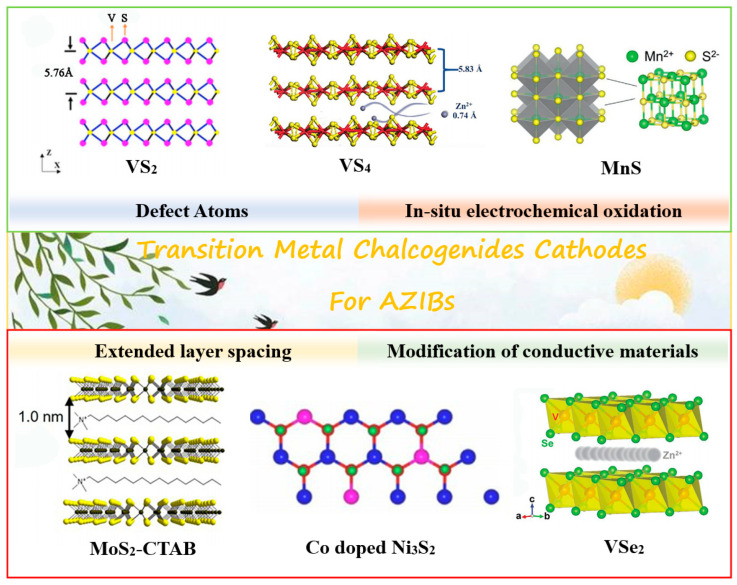
The classification of typical TMCs cathodes and the strategies of modification.

Two-dimensional (2D) TMCs are composed of transition metals (M = V, Ni, Mo, W and Mn, etc.) and chalcogen elements (X = S, Se, Te) with tunable electrical properties from semiconductors to metals. They is widely studied in the field of energy storage and conversion field [24,27,28]. In addition, graphene-like 2D layered TMCs are highly advantageous for battery applications because of their large specific surface area, which can significantly increase the contact area between the active material and the electrolyte [29]. Figure 1 illustrates the crystal structures of various TMCs materials. Their non-bonding properties allow the insertion of atoms, ions and molecules. The design strategies of TMCs materials mainly include defect engineering, hybridization, phase modulation, and in situ electrochemical oxidation. The main focus is to widen the interlayer space, improve the electrode conductivity, and accelerate the electrochemical kinetic process. We will categorize the strategies of structural design and zinc storage capabilities of various cathode materials in the following sections.

### 2.1. VS_2_ and VS_4_

**Figure 2:**
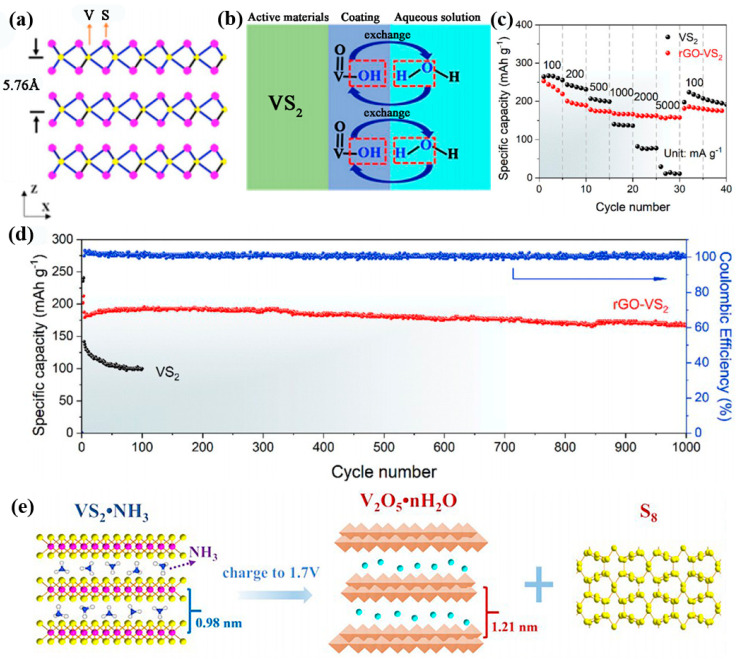
The electrochemical performance of VS_2_ electrodes. (**a**) Atomic structure of VS_2_ materials. (**b**) The working mechanism of cathode. Reproduced with permission [31]. Copyright 2019, Elsevier B.V. (**c**) Rate capability of rGO–VS_2_ and VS_2._ (**d**) Cycling performance of these two electrodes. Reproduced with permission [32]. Copyright 2020, Elsevier B.V. (**e**) Zn^2+^ storage mechanism. Reproduced with permission [35]. Copyright 2021, Elsevier Inc.

The hexagonal-structured vanadium disulfide (VS_2_) is a member of the TMC family. It owns a layered structure with a layer spacing of 5.76 Å [30]. The S-V-S layers rely on weak van der Waals interactions (Figure 2a). Hence, VS_2_ materials have become an attractive host for Zn ion insertion/extraction. Nevertheless, VS_2_ is unstable in aqueous solutions, leading to severe capacity decay during cycling. From the aspect of modulating the material structure, coating is considered to be effective strategy. Pu et al. prepared rose-shaped VS_2_ encapsulated with a hydrophilic VOOH coating using a one-pot hydrothermal route [31]. The assembled cells maintained 82% capacity after 400 cycles. The rate capability and long cycle life of the optimized sample is significantly improved compared to the VS_2_ sample, which is attributed to the O–H in VOOH. From Figure 2b, its presence not only enhances the wetting of the electrode and electrolyte, but also prevents the dissolution of the main materials. Fan’s group prepared ultrathin VS_2_ nanosheets grown on graphene sheets (rGO–VS_2_) by a solvothermal strategy [32]. The rGO offers a large specific surface area and excellent electrical conductivity. In addition, the close contact of VS_2_ nanosheets with rGO can effectively prevent the dissolution and corrosion of the host materials. It ensures the high stability of the electrode in long-term cycling. Thus, Zn/rGO–VS_2_ cells can deliver a large specific capacity (238 mAh g^−1^ at 0.1 A g^−1^) and excellent rate performance (190 mAh g^−1^ at 5 A g^−1^, Figure 2c). After 1000 cycles of charging and discharging, it can still maintain 93.3% of the initial capacity, as shown in Figure 2d.

The N dopant provides high affinity for the transition metals, so it is possible to form a strong coupling between the host material and the N-doped carbon. This contributes to accelerate the interfacial electron transfer and reduces cycling-induced stress and volume changes. Liu and co-workers prepared spun VS_2_ materials on a N-doped carbon layer (VS_2_@N-C) by an in situ hybridization strategy [33]. This strategy ensures a strong interfacial interaction between the active material and the N-doped carbon. It promotes the enhancement of electrochemical kinetics and cycling stability of the cathode. The optimal electrode possesses a capacity of 203 mAh g^−1^ at 0.05 A g^−1^. Based on the Zn ion insertion and extraction mechanism, the cells can obtain a capacity of 144 mAh g^−1^ after 600 cycles. Liu’s group synthesized 1T-VS_2_ colloidal nanospheres assembled from nanoflakes [34]. By controlling the charge cutoff voltage, a number of the Zn ions were trapped in the interlayer of the structure after the initial charge/discharge cycle. These “dead Zn” act as “pillars” to ensure the stability of the layered structure of VS_2_. After 2000 cycles, the cells maintained the capacity retention of 86.7%. This unique layered structure increases the conductivity due to the presence of carbon and oxygen groups on the surface, facilitating the penetration of the aqueous electrolyte and providing more active sites.

**Figure 3:**
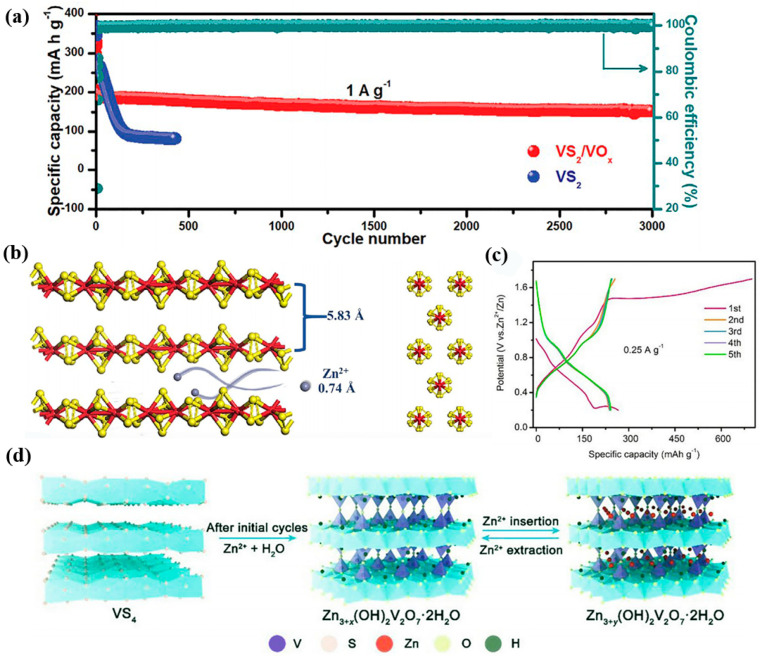
The energy storage mechanism and cycling performance. (**a**) The comparison of cycling performance at 1 A g^−1^. Reproduced with permission [36]. Copyright 2020 Wiley-VCH GmbH. (**b**) The crystal structure of VS_4_ materials. Reproduced with permission [37]. Copyright 2020, The Royal Society of Chemistry. (**c**) The charge–discharge curves at 0.25 A g^−1^. (**d**) Schematic of the energy storage mechanism of Zn–VS_4_/CNTs batteries. Reproduced with permission [41]. Copyright 2021, Elsevier B.V. and Science Press.

In order to further improve the stability of VS_2_ electrodes in an aqueous electrolyte. Yang et al. employed an in situ electrochemical oxidation approach to enhance the interlayer space of vanadium disulfide (VS_2_NH_3_) hollow spheres [35]. This large layer spacing (1.21 nm) is favorable to the improvement of zinc ion storage capacity. The VS_2_NH_3_ samples transform into a porous structured V_2_O_5_·nH_2_O phase during the first charging cycle (Figure 2e). It enhances the active sites and is conducive to a rapid electrochemical kinetic process. This derived electrode maintains a high capacity at 3 A g^−1^ even after 2000 cycles. Similarly, Du and co-workers proposed the formation of VS_2_/VO_x_ heterostructures by in situ electrochemical induction [36]. When the sample is charged to 1.8 V, the morphology of the composite changes from rose-like shape to sheet-like one. This structure can withstand the volume expansion caused by repeated cycles. Compared to the pure Zn–VS_2_ cell, the Zn–VS_2_/VO_x_ one demonstrates a cycling stability of 3000 cycles at 1 A g^−1^ with an improved working potential of 0.25 V (Figure 3a). This strategy of combining highly conductive sulfides and excellent chemically stable oxides leads to an enhancement of the Zn^2+^ storage capacity of the VS_2_/VO_x_ cathode.

VS_4_ is a material with a one-dimensional (1D) atomic chain structure. When compared with the VS_2_ material, it possesses many S atoms with layer spacing up to 5.83 Å, as shown in Figure 3b. It indicates that VS_4_ may show excellent zinc storage capacity. Zhu et al. prepared VS_4_ materials as cathodes via a hydrothermal route [37]. Density functional theory (DFT) calculations demonstrate that the electrode is capable of storing zinc ions up to a maximum specific capacity of 262 mAh g^−1^. Then, the cell can deliver a specific capacity of 310 mAh g^−1^ at 0.1 A g^−1^. It is higher than the theoretical capacity, which could be due to the additional absorption capacity. Furthermore, the absence of additional by-product generation suggests that the energy storage of material follows a zinc-ion embedding/de-embedding mechanism. The construction of heterostructures is also an attractive strategy. In theory, the intrinsic zinc storage capacity of the cathode can be effectively enhanced by building a heterostructure with sufficient interfaces and grain boundaries. Fang’s group designedVS_4_/V_2_O_3_ heterostructures with a high specific surface area [38]. The assembled battery shows a capacity of 163 mAh g^−1^ at 0.1 A g^−1^. As a contrast, the single electrode presents inferior electrochemical performance. This demonstrates that the optimized heterogeneous material can boost the energy storage capacity.

It is also an effective strategy for improving electrochemical kinetics by compositing with highly conductive materials. Qin and co-workers synthesized VS_4_ composite material immobilized on reduced graphene oxide (VS_4_@rGO) as a cathode [39]. This synergistic effect enables the VS_4_@rGO electrode to reach a capacity of 180 mAh g^−1^ (1 A g^−1^) after 165 cycles with a capacity retention of 93.3%. Nevertheless, the above-mentioned electrochemical performance is still unsatisfactory. Chen and co-workers optimized the morphology of the VS_4_@rGO composites to achieve a specific capacity of 450 mAh g^−1^ at a current density of 0.5 A g^−1^ when used as cathodes [40]. Moreover, the capacity of 313.8 mAh g^−1^ was maintained at high current densities (10 A g^−1^). It indicates that the batteries possess an excellent rate capability. It is noteworthy that a new phase Zn_3_(OH)_2_V_2_O_7_·2H_2_O (ZVO) appears during charging. The following reactions may occur in the electrode material:

VS_4_ + xZn^2+^ + 2xe^−^ ↔ Zn_x_VS_4_ (1)

VS_4_ + 11H_2_O + 3Zn^2+^ → Zn_3_(OH)_2_V_2_O_7_·2H_2_O + 8S + 16H^+^ + 10e^−^ (2)

After that, it transforms into the ZnV_3_O_8_ phase during the long cycles, which is associated with the subsequent capacity decay. Gao et al. reported a flower-like VS_4_/CNTs cathode with an abundant mesoporous structure, which effectively shortens the diffusion path of zinc ions [41]. In Figure 3c, when the first cycle is charged to 1.7 V, the charging curve undergoes a slow upward trend, which implies a phase transition process. Figure 3d further confirms that the mechanism of the phase change reaction of VS_4_ with zinc pyrovandate (Zn_3+x_(OH)_2_V_2_O_7_·2H_2_O). The results show that the Zn–VS_4_/CNTs batteries possess a reversible capacity of 265 mAh g^−1^ (0.25 A g^−1^) and a good rate performance in the potential range from 0.2 to 1.7 V. Although the energy storage capacity has been significantly improved by modification of the electrode material, the inevitable phase change during the reaction process still hinders the cycle life. This may be related to the high charging voltage.

### 2.2. MoS_2_

**Figure 4:**
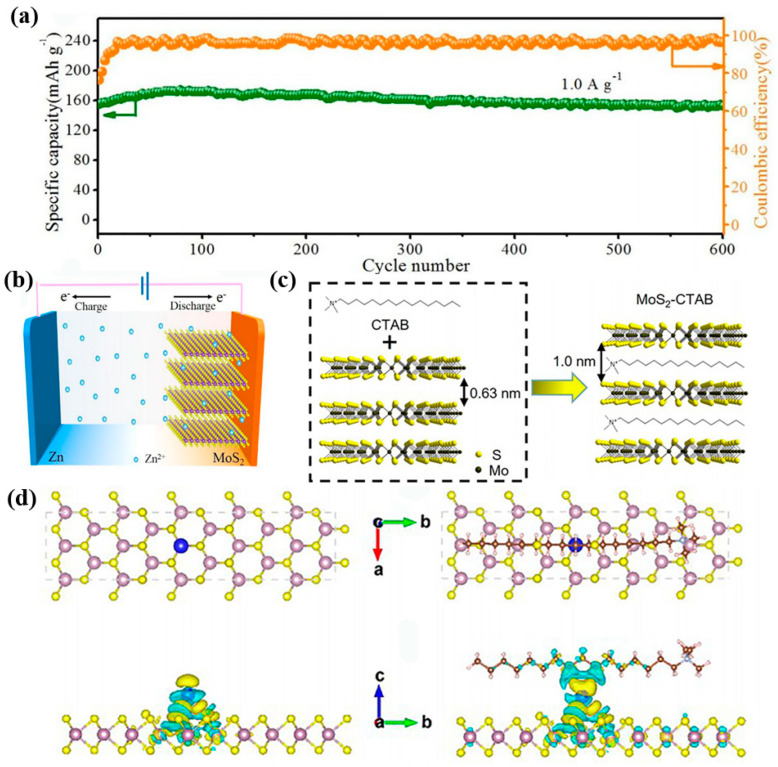
The modification of MoS_2_ materials and DFT calculation. (**a**) Cycling stability of Zn/E–MoS_2_ batteries at 1.0 A g^−1^. (**b**) Schematics of the mechanism of batteries. Reproduced with permission [45]. Copyright 2018, Elsevier B.V. (**c**) Schematic of the MoS_2_–CTAB superlattice nanosheets and the d-spacing of the samples. (**d**) The charge density of pristine MoS_2_ and MoS_2_–CTAB nanosheets during the insertion of Zn^2+^. Reproduced with permission [51]. Copyright 2022, American Chemical Society.

MoS_2_ is a typical 2D-layered structure bound by weak van der Waals forces [42,43,44]. However, the ionic radius of hydrated Zn^2+^ is 0.43 nm, which places high demands on the interlayer space of the host materials. To enhance the reaction kinetics of Zn ion insertion and extraction, Li et al. extended the interlayer spacing of the (002) plane of MoS_2_ nanosheets from 0.62 nm to 0.70 nm [45]. Due to the addition of glucose, an amorphous carbon layer is wrapped on the surface of MoS_2_. This facilitates the alleviation of volume expansion and promotes charge transfer. From Figure 4a, the specific capacity of the batteries can be maintained at 164.5 mAh g^−1^ after 600 cycles. In Figure 4b, the charge storage mechanism can be described as follow:

Cathode: xZn^2+^ + x2e^−^ + MoS_2_ ↔ Zn_x_MoS_2_ (3)

Anode: Zn^2+^ + 2e^−^ ↔ Zn (4)

In addition, a flexible solid-state Zn/E-MoS_2_ cell was further assembled using the starch/polyacrylamide (PAM) polymer electrolyte. Under different mechanical strengths, the cell still can maintain a stable charge/discharge process.

Due to the diversity of coordination of Mo and S atoms, MoS_2_ can show a semiconductor phase with a triangular prismatic structure (2H phase) and a metallic phase with an octahedral structure (1T phase). 1T-phase MoS_2_ possesses higher electrical conductivity and better hydrophilicity than 2H-phase ones [46,47]. Therefore, the material is also a promising electrode for zinc storage. Huang et al. synthesized a 1T-phase MoS_2_ nanosheet grown directly on reduced graphene oxide (rGO) scaffolds [48]. The addition of the rGO scaffold can serve to stabilize the 1T phase and reduce the possibility of phase transition during zinc ions insertion/extraction. In addition, it can improve the electrical conductivity, thus shortening the diffusion path of zinc ions. The initial discharge capacity of the 1T-MoS_2_/rGO heterogeneous electrode is 108.3 mAh g^−1^, and the cell maintains a capacity retention of 88% after repeated charge/discharges of 1000 times.

Tang’s group synthesized N-doped 1T MoS_2_ nanoflowers assembled from ultrathin nanosheets by a one-step hydrothermal sulfidation of Mo-based organic framework (MOF) precursors [49]. The introduction of defects effectively widens the interlayer spacing and increases the number of sulfur vacancies as well as the hydrophilicity of the sample. Zn/N-doped 1T MoS_2_ batteries deliver a capacity of 149.6 mAh g^−1^ (0.1 A g^−1^). The capacity retention is up to 89.1% after 1000 cycles at 3 A g^−1^. Additionally, the electrochemical performance was studied for the difference in area mass loading of the electrodes. The area capacity shows an outstanding performance when the area loading reaches 1.701 mg cm^−2^. This implies that the electrode presents excellent rate capacity even at high loadings. Liu and co-workers reported MoS_2_ nanosheets with different phase contents as cathode materials [50]. Among them, the MoS_2_ nanosheet electrode with 1T phase of content around 70% presents favorable long-term cycling stability. This indicates that the presence of metallic 1T phase favors the ion and charge transfer.

Apart from composite with conductive materials, combination with organic molecules also promotes the increase of zinc ion storage capacity. Yao et al. designed a 2D MoS_2_/C_19_H_42_N^+^ (CTAB) organic–inorganic superlattice structure (MoS_2_–CTAB) as a cathode [51]. This unique structure can significantly enlarge the interlayer spacing (1.0 nm) of the host materials (Figure 4c). In addition, the stable electrode structure can accommodate the expansion and contraction of Zn^2+^ within the host structure. The loading mass of the active material is a very important parameter for the evaluation of the specific capacity and energy density of the cell. The areal capacity of the battery increases with the area mass loading in a potential window of 0.2–1.3 V. Figure 4d demonstrates the optimal adsorption position of Zn ions at the pure MoS_2_ and modified MoS_2_ electrodes by DFT calculations. In the former structure, the Zn ion prefers to adsorb at the top site of the Mo atom with a corresponding energy of −0.31 eV, but the charge accumulation of adsorbed Zn with adjacent S atoms suggests a strong electrostatic interaction between Zn and the host material. In the latter one, the adsorption energy of Zn ion at the same site is −0.25 eV, and the distance between Zn and its three neighboring S atoms remains almost unchanged relative to the original electrode.

### 2.3. MnS

In recent years, many efforts have been made in Mn-based oxide cathodes [52]. For instance, Minakshi et al. compared the cathodic behavior of electrolytic manganese dioxide (EMD) and chemically prepared battery-grade manganese dioxide (BGM) in a lithium hydroxide (LiOH) electrolyte [53]. The EMD cell demonstrated stable discharge/charge cycles compared to the BGM. Wang’s group designed nanocrystal line structures of MnO_2_ materials with particle sizes typically less than 10 nm [54]. This structure confers some electrode/electrolyte contact interfaces. Therefore, the Zn/MnO_2_ cell delivers a capacity of 260 mAh g^−1^ at 1.3 C.

**Figure 5:**
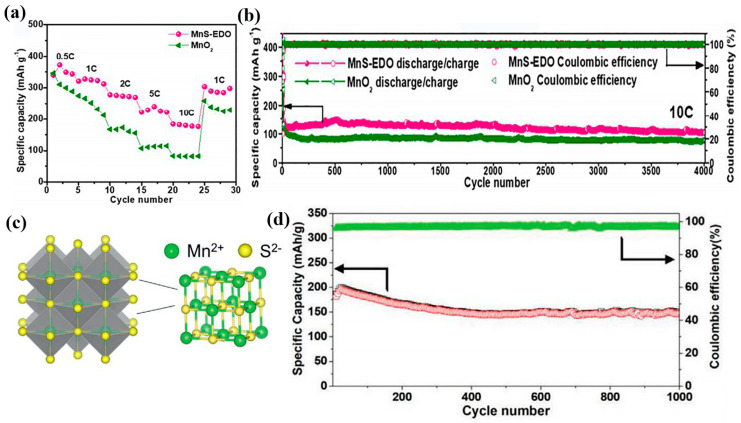
The long-term cycle performance of MnS electrodes. (**a**) Rate capability of the two electrode. (**b**) The long-term cycling at 10 C. Reproduced with permission [56]. Copyright 2020, Elsevier Ltd. (**c**) Crystal structure model of α-MnS; (**d**) Cyclic stability in 2 M ZnSO_4_ and 0.1 M MnSO_4_ aqueous electrolyte. Reproduced with permission [58]. Copyright 2021, Wiley-VCH GmbH.

However, their rate capability and cycle stability cannot meet the current high-capacity energy storage requirements. In addition, Zn ions show strong electrostatic interactions with the Mn oxide lattice, leading to large energy barriers for Zn^2+^ migration [55]. Chen et al. reported the transformation of α-MnS materials into high-performance manganese oxide cathodes (MnS–EDO) by in situ electrochemical oxidation [56]. Compared to α-MnO_2_, this electrode generated more defects and vacancies after structural reconfiguration. This indicates a rapid electrochemical kinetic process. The Zn/MnS–EDO cell shows a high specific capacity of 335.7 mAh g^−1^ with capacity retention close to 100% and reversible rate performance (Figure 5a). In addition, it can undergo a repeated charge/discharge process of 4000 cycles, as shown in Figure 5b.

To further enhance the electrical conductivity of MnS, Ma and co-workers designed a MnS and rGO composite material. This synergistic effect effectively improves the Zn storage capacity of the electrode material [57]. MnS possesses various phase types: α-MnS, β-MnS and γ-MnS. Both β- and γ-phases are sub-stable and readily transform to the stable rock salt structure α-MnS (Figure 5c). Jiang et al. fabricated flexible zinc ion microcells with MnS as cathodes and guar gels as the quasi-solid electrolyte by etching soft templates on various substrates [58]. The cells prepared on PET substrates deliver an area-specific capacity of 178 μAh cm^−2^. After 1000 cycles, they can maintain a capacity of 150 mAh g^−1^ at 1 A g^−1^ in Figure 5d. Moreover, the area energy density can reach 322 μWh cm^−2^ at a power density of 120 μW cm^−2^. In addition, this quasi-solid-state cell shows excellent flexibility with almost no significant capacity degradation when the device is bent at multiple angles.

### 2.4. VSe_2_

Among numerous TMCs, vanadium diselenide (VSe_2_) presents a typical layered structure with a sandwich-like Se–V–Se connected by van der Waals interactions [59]. The materials possess a layer spacing of 6.11 Å, which can provide sufficient transport channels and active sites for the intercalated ions [60]. Moreover, the strong electronic coupling force between adjacent V^4+^–V^4+^ endows it with metallic properties. Thus, it shows great potential in terms of energy storage [61,62]. For instance, Alshareef’s group explored the energy storage capacity of VSe_2_ materials in different alkali metal batteries [63]. The morphology of VSe_2_ nanosheets can be modulated using *N*-methylpyrrolidone (NMP) solvent, and the electrochemical performance of the samples is further improved by in situ carbon coating. The electrodes can provide a specific capacity of 768 mAh g^−1^ (lithium storage) and 571 mAh g^−1^ (sodium storage), respectively.

**Figure 6:**
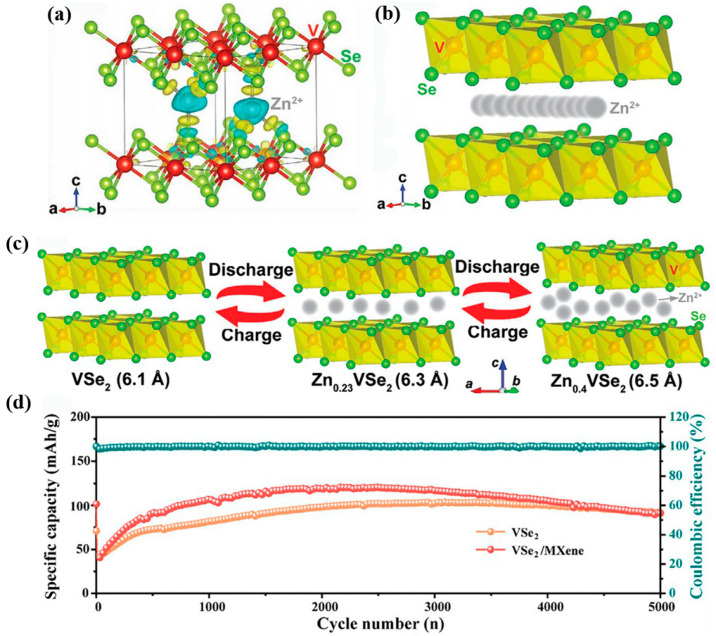
The kinetic process and cycle capability of a VSe_2_-based Zn battery. (**a**) The schematic of charge density map after zinc-ion intercalation. (**b**) The optimal diffusion pathway of zinc ions. (**c**) Schematic of the two-step Zn^2+^ intercalation/de-intercalation process. Reproduced with permission [64]. Copyright 2020, Wiley-VCH GmbH. (**d**) Long-cycles performance at 5 A g^−1^. Reproduced with permission [65]. Copyright 2022, Elsevier Inc.

Recently, VSe_2_ materials also show attractive performance in zinc storage. Wu et al. designed an ultrathin VSe_2_ nanosheet as a cathode [64]. The assembled cells maintain an initial capacity of 80.8% after 500 cycles. This durable cycling stability is attributed to their fast Zn^2+^ diffusion kinetic process and durable cycling stability. The local charge density map in Figure 6a shows a decrease in charge around the Zn ion and an increase in charge around the Se and some V sites. This demonstrates that the intercalated Zn is bonded to the Se ligand. From Figure 6b, the migration barrier for the optimal diffusion pathway of Zn ions is 0.91 eV, which corresponds to a fast ion migration rate. Based on the Zn ion insertion and extraction mechanism (Figure 6c), the cell possesses an energy density of 107.3 Wh kg^−1^ at a power density of 81.2 W kg^−1^. Cai and co-workers synthesized homogeneous flower-shaped VSe_2_ spheres using MXene as a support [65]. The specific capacity of Zn–VSe_2_/MXene cells was higher than the initial capacity after 2000 cycles at 5 A g^−1^ in the voltage window of 0.2–1.6 V (Figure 6d). This may be attributed to the generation of a Zn_0.25_V_2_O_5_ (ZVO) phase. With repeated discharge/charging, the generation and accumulation of ZVO phase provides a continuous capacity contribution to the battery.

### 2.5. Ni_3_S_2_

Nickel sulfide (Ni_3_S_2_) is a promising electrode material with the advantages of high activity and theoretical capacity, excellent stability, and low cost [66,67]. However, Ni_3_S_2_ materials perform inferiorly in cycling stability and specific capacity when they are used as a cathode for AZIBs. Structural defects are an approach to modulate the crystal structure of the materials [68]. In addition, the introduction of defects can increase the electrical conductivity and carrier density, effectively improving the electrochemical activity of the electrode materials [69].

For instance, Tong et al. doped highly reactive Co ions in Ni_3_S_2_ nanocones by atomic layer deposition (ALD) and the hydrothermal method [70]. A Co12-Ni_3_S_2_/NF (C12NS) sample was used as the cathode; the hydrogel electrolyte is immersed in 5 M KOH/0.1 M Zn(AC)_2_ solution to assemble a quasi-solid-state flexible cell. The reactivity of the electrodes is increased due to the Co doping and sulfation. The specific capacity of the activated electrode is about four times higher than that before activation. This is mainly due to the adsorption of OH radicals from the alkaline electrolyte on the sulfur sites of the Co-doped Ni_3_S_2_. The reaction mechanism can be described as:

Cathode: Co-Ni_3_S_2_ + 2OH^−^ ↔ Co-Ni_3_S_2_(OH)_2_ + 2e^−^ (5)

Co-Ni_3_S_2_(OH)_2_ + 3OH^−^ ↔ Co-Ni_3_S_2_O_2_OH + 2H_2_O + 3e^−^ (6)

Anode: Zn[(OH)_4_]^2–^ + 2e^−^ ↔ Zn + 4OH^−^ (7)

**Figure 7:**
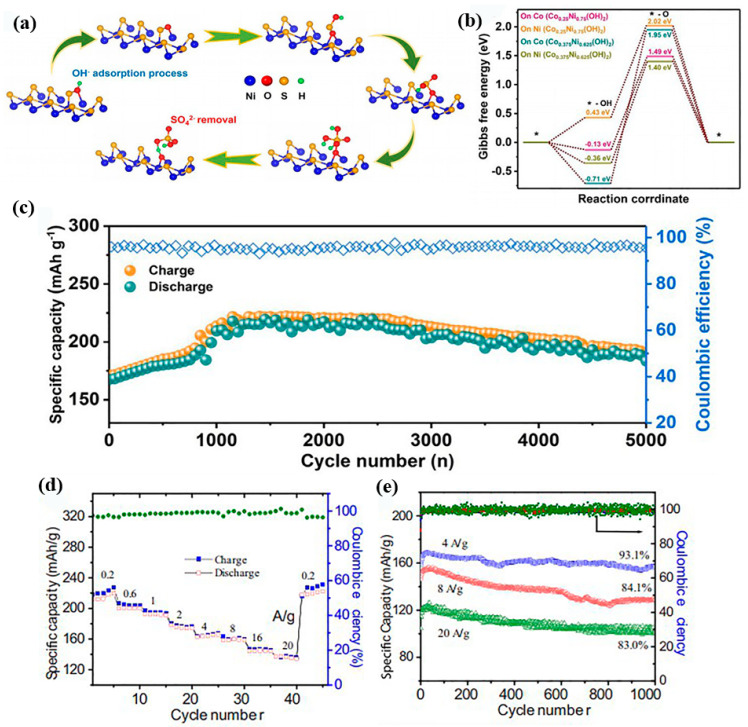
The electrochemical performance of Zn/Ni_3_S_2_ batteries. (**a**) Schematic of nickel sulfide-adsorbing hydroxide radicals and being oxidized to form nickel hydroxide and sulfate radicals. (**b**) Gibbs free energies of Zn/Ni_3_S_2_ batteries. (**c**) The cycling stability of the flexible ZC12NS battery. Reproduced with permission [70]. Copyright 2021, Elsevier B.V. and Science Press. (**d**) Rate capability. (**e**) The long-term performance. Reproduced with permission [71]. Copyright 2021, Elsevier B.V.

During the continuous adsorption of hydroxide radicals, sulfur and oxygen coexist. The formed oxygen–sulfur bonds enhance the electrochemical performance of the electrode material, as shown in Figure 7a. The Co-doped electrodes present a low energy barrier. It indicates that they can achieve a fast Zn ion migration (Figure 7b). In Figure 7c, the capacity retention of Co12-Ni_3_S_2_/NF electrode after 5000 cycles is up to 90%.

In addition to the defect strategy, Wang’s group prepared Ni/Ni_3_S_2_ nanocomposites with large specific surface areas using Ni–ZIF MOFs as precursors through a simple medium temperature solid–gas phase reaction [71]. The assembled batteries with 6 M KOH-0.6 M ZnO electrolyte possess an excellent rate capability (Figure 7d). They can maintain a capacity retention of 83% after 1000 cycles at 20 A g^−1^ (Figure 7e). Additionally, an energy density of 379 Wh kg^−1^ can be obtained at 340 W kg^−1^ at a high output voltage (1.7 V). Table 1 summarizes the electrochemical performance of TMC cathodes. It can be observed that these materials present some disadvantages, mainly in terms of limited voltage window, low specific capacity and inferior cycle life. In addition, the modification tactics lead to a large difference in the electrochemical performance of the electrodes. This demonstrates that an appropriate strategy can enhance the structural stability as well as the cycle life of the cells. The VS_4_ composite shows significant advantages with a cycle life of 3500 cycles with a stable charge/discharge process. It can be noted that the electrolytes used in these batteries are varied. The high concentration of electrolyte contributes to the increase in capacity. Therefore, the choice of electrolyte is also crucial in future work to affect the electrochemical performance of the Zn/TMCs batteries.

## 3. Summary and Outlook

AZIBs have attracted a considerable attention as an alternative to LIBs with the features of being inexpensive, environmentally friendly, resource-rich, and having high theoretical capacity. However, current cathode materials are still hampered by their inferior conductivity, slow kinetics, structural instability, and dissolution of active substances. Among them, TMCs possess a unique layered structure and a large layer spacing and high conductivity, which facilitate the transfer of ion carriers. First, the high specific surface area provides many electrochemically active sites and short ion transfer paths. Secondly, its excellent electrical conductivity ensures fast electron transfer. Finally, the open-layer structure favors the embedding of electrolyte ions and reduces the volume change. We summarized the research advances of TMCs as electrode materials in recent years. The main focus was on their modification strategies and the improvement of zinc storage capacity. Reasonable modification strategies are beneficial for the improvement of energy storage capability. Nevertheless, the goal of commercialization is still not reached. More efforts may be required to try to shorten this gap.

The electrochemical performance of TMCs materials, such as specific capacity and long cycle performance, still needs to be improved. There are several strategies for considering directions for this: (1) introducing defects (vacancies or doping) in electrode materials. It can add many active sites and conductivity to effectively increase the capacity of the battery. (2) Combining high conductivity and specific surface area materials such as MOFs and rGO. MOFs possess rich pore structures. The calcination of MOFs precursors is an effective method to prepare metal compound/carbon composites. (3) Expanded layer spacing. The increase of layer spacing is beneficial to the rapid shuttling of zinc ions, which can effectively improve the electrochemical kinetics.

**Figure 8:**
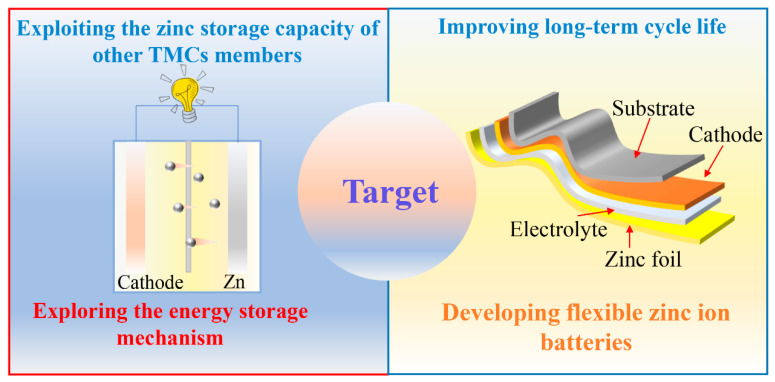
The future target of TMCs cathodes for AZIBs.

Although some TMCs have been employed for energy storage, there are few materials with desired zinc storage capability. Therefore, it is still necessary to explore the unexploited cathode materials (Figure 8). We should place our eyes on the study of materials such as WS_2_, MoSe_2_, etc., and focus on the design of effective modification strategies. Additionally, the mechanism of electrochemical energy storage is still unclear. Advanced in situ characterization techniques are beneficial to explore the evolution of the structure of electrode materials during charging and discharging as well as elemental valence changes.

In addition, the structural stability of electrode materials is closely associated with their electrochemical performance. The cycling process may generate irreversible phase changes and side reactions, which lead to structural collapse or capacity loss. The modulation of heterogeneous interfaces may suppress the occurrence of side reactions. Finally, flexible electronic devices have attracted much attention because of their high safety, portability, and wearable features. There are few reports related to flexible devices for AZIBs. They should focus on flexible substrates and cathodes with certain mechanical flexibility, and stable solid-state electrolytes with high ionic conductivity.

## Figures and Tables

**Table 1:** The electrochemical performance of TMC cathodes.

Cathodes	Anodes	Voltage (V)	Electrolyte	Capacity (mAh g^−1^)	Cycle Stability (mAh g^−1^, Cycles, A g^−1^)	Ref.
VS_2_@VOOH	Zinc foil	0.4–1.0	3 M ZnSO_4_	165 (0.1 A g^−1^)	91.4, 400, 2.5	[31]
VS_2_ and N-doped carbon	Zinc foil	0.2–1.8	3 M Zn(CF_3_SO_3_)_2_	203 (0.05 A g^−1^)	144, 600, 1	[33]
VS_2_/VO_x_	Zinc foil	0.1–1.8	25 M ZnCl_2_	260 (0.1 A g^−1^)	75%, 3000, 1	[36]
VS_4_	Zinc foil	0.2–1.6	1 M ZnSO_4_	310 (0.1 A g^−1^)	110, 500, 2.5	[37]
VS_4_/V_2_O_3_	Zinc foil	0.3–1.2	3 M Zn(CF_3_SO_3_)_2_	163 (0.1 A g^−1^)	-	[38]
VS_4_@rGO	Zinc foil	0–1.8	1 M Zn(CF_3_SO_3_)_2_	450 (0.5 A g^−1^)	82%, 3500, 10	[40]
MoS_2_	Deposited zinc on carbon cloth	0.5–1.5	2 M ZnSO_4_	202.6 (0.1 A g^−1^)	164.5, 600, 1	[45]
MoS_2_-CTAB	Zn anode plated on carbon paper	0.2–1.3	3 M ZnSO_4_	181.8 (0.1 A g^−1^)	92.8%, 2100, 10	[51]
MnS	zinc powder	0.9–1.95	2 M ZnSO_4_ and 0.1 M MnSO_4_	297 (0.1 A g^−1^)	150, 1000, 1	[58]
VSe_2_	Zinc foil	0.2–1.6	2 M ZnSO_4_	131.8 (0.1 A g^−1^)	80.8%, 500, 0.1	[64]
Ni/Ni_3_S_2_	Zinc foil	1.3–1.9	6 M KOH and 0.6 M ZnO	220 (0.2 A g^−1^)	93.1%, 1000, 4	[71]
